# Biomechanical comparison of the anterior reverse PHILOS and locking compression plate extra-articular distal humerus plates for extra-articular distal humeral fractures

**DOI:** 10.3389/fbioe.2025.1672989

**Published:** 2025-10-01

**Authors:** Apipop Kritsaneephaiboon, Sujin Wanchat, Thammaphong Khongkanin, Atichart Kwanyuang, Satta Srewaradachpisal, Kantapon Dissaneewate, Wich Orapiriyakul

**Affiliations:** ^1^ Department of Orthopedics, Faculty of Medicine, Prince of Songkla University, Songkhla, Thailand; ^2^ Department of Mechanical Engineering, Faculty of Engineering at Sriracha, Kasetsart University, Sriracha, Chonburi, Thailand; ^3^ Faculty of Medicine Siriraj Hospital, Golden Jubilee Medical Center, Mahidol University, Nakhon Pathom, Thailand; ^4^ Institute of Biomechanical Engineering, Faculty of Medicine, Prince of Songkla University, Songkhla, Thailand; ^5^ Department of Mechanical Engineering, Faculty of Engineering, Prince of Songkla University, Songkhla, Thailand

**Keywords:** PHILOS, distal humeral fracture, extra-articular, biomechanical study, internal-fixation method

## Abstract

**Background:**

The locking compression plate extra-articular distal humeral plate (EADHP) is an anatomically pre-contoured plate that is used for extra-articular distal humeral fractures. However, there is currently no standard criterion for the internal fixation of this type of fracture. Moreover, the anterior reverse proximal humeral internal locking system (PHILOS) plate (ARPP) has been clinically applied as a new internal-fixation plate without testing in biomechanical studies. We aimed to compare the biomechanical properties of ARPP and EADHP for the definitive fixation of extra-articular distal humeral fractures.

**Methods:**

Eighteen composite humerus bones were cut at the distal humerus using an electrical saw to generate a fracture gap. Internal fixation via the ARPP or EADHP was performed following standard techniques. An Instron testing machine (Instron 8872) was used to evaluate biomechanical properties by applying bending torque, axial force, and torsional torque.

**Results:**

Fixations with both ARPP and EADHP could withstand forces that exceeded the physiological forces (200 N). Under axial compression, ARPP constructs demonstrated greater stiffness (668.9 ± 120.7 N/mm vs 171.2 ± 45.4 N/mm) and higher maximal load-to-failure (2,092.6 ± 305.2 N vs 907.0 ± 56.5 N) compared with EADHP, although these differences were not statistically significant. During anterior bending, ARPP provided significantly higher stiffness (17.8 ± 2.0 N/mm vs 13.9 ± 1.0 N/mm, *p* = 0.041), whereas EADHP showed a higher but non-significant load-to-failure. Under torsional loading, ARPP tended to exhibit greater stiffness in both external and internal rotation, as well as higher load-to-failure (31.1 ± 0.8 N m vs 26.0 ± 4.4 N m), but without statistical significance.

**Conclusion:**

ARPP demonstrated superior bending stiffness compared with the EADHP, while both constructs performed equivalently in axial compression and torsion. Therefore, ARPP can serve as an alternative internal-fixation method for extra-articular distal humeral fractures.

## 1 Introduction

Extra-articular distal humeral fracture (EADHF) constitutes a technically challenging condition in orthopaedic trauma practice owing to its unique bone geometry and frequent presentation of complex fracture pattern. The optimal treatment of this fracture remains unelucidated as yet (Ring et al., 2016). Although nonoperative treatment provides a high union rate without increased risk of radial nerve injury, as well as excellent functional outcomes, the difficulty in controlling fracture alignment using a functional brace potentially results in a late angular deformity, especially in comminuted fractures, and this remains a major drawback (Sarmiento et al., 1990; Ekholm et al., 2006). A more predictable fracture alignment and potentially quicker return of function could be achieved by operative treatment; however, undesirable consequences, such as iatrogenic radial nerve injury, infection, and a second operation, comprise the main disadvantages of this treatment (Jawa et al., 2006).

The goals of the operative treatment of EADHF are to restore fracture alignment and obtain stable fixation to permit early mobility of the elbow, which is crucial for ensuring satisfactory functional outcomes (Self et al., 1995; Capo et al., 2014). Currently, open reduction and internal fixation with posterior plating are considered reliable surgical options for distal diaphyseal humeral fractures. The extra-articular distal humerus locking compression plate (EADHP) is a device specifically designed for fixing low diaphyseal humeral fractures via the posterior approach (Capo et al., 2014; Kharbanda et al., 2017; Ali et al., 2018). The advantages of this pre-contoured plate include providing angular stability, ease of implant placement on the flat surface of the posterolateral column of the distal humerus and allowing for a greater number of screws for distal fixation (Capo et al., 2014; Kharbanda et al., 2017; Ali et al., 2018). Nevertheless, several complications could arise from posterior plating of the humerus, including decreased muscular strength when using the triceps-splitting approach (Illical et al., 2014), increased risk of radial nerve injury, and symptomatic hardware irritation at the posterior elbow that may necessitate implant removal (Capo et al., 2014; Gallucci et al., 2015; Kharbanda et al., 2017; Ali et al., 2018; Jiamton et al., 2019).

The proximal humeral internal locking system (PHILOS) is commonly used for proximal humeral fixation. Recent clinical series by Park et al. (2015), Sohn and Shin (2019), Jitprapaikulsan et al. (2020) and Fu et al. (2025) presented an alternative technique for the fixation of EADHFs using the PHILOS in reverse orientation via the anterior approach. This technique demonstrated satisfactory functional results in terms of both the range of motion of the elbow and fracture union rate, without increasing the risk of radial nerve injury and plate breakage. The anterior reverse PHILOS plate (ARPP) has a well-conformed shape between the plate and anterior surface of the distal humeral metaphysis and allows multiple multidirectional screw fixations in a short distal fragment.

This study was conducted with an aim to compare the stiffness and failure load of ARPP with those of EADHP in EADHFs. We hypothesised that there would be no difference in the stiffness or load-to-failure values between these two internal fixations.

## 2 Materials and methods

### 2.1 Specimen preparation

**FIGURE 1:**
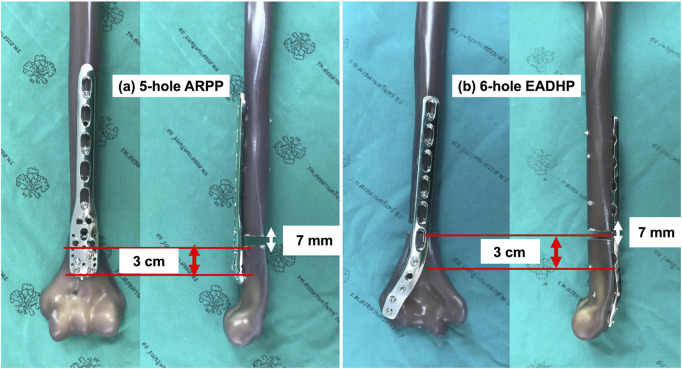
Preparation of the fracture model with a 7-mm osteotomy gap created 3 cm proximal to the olecranon fossa. **(a)** The 5-hole ARPP and **(b)** the 6-hole EADHP, each fixed to the anterior and posterior cortices with three bicortical locking screws in the proximal fragment and four bicortical locking screws in the distal fragment.

Eighteen large, left, fourth-generation, composite humeri (Item #3404–4, Sawbones Corporate Headquarters, Washington, USA) that simulated the structural and material properties of cadaveric bones were used in this study. A fracture model of multifragmentary extra-articular diaphyseal fractures of the distal humerus (type 12-C3 according to the Arbeitsgemeinschaft für Osteosynthesefragen/Orthopaedic Trauma Association (AO/OTA) classification) was selected. A 7-mm osteotomy gap, starting 3 cm proximal to the superior margin of the olecranon fossa, was created using an oscillating bone saw to simulate metaphyseal comminution as described previously (Self et al., 1995; Korner et al., 2004; Tejwani et al., 2009; Kollias et al., 2010; Shimamura et al., 2010). Thus, there was no bone contact between the fragments during the mechanical testing (Figures 1a,b).

### 2.2 Osteosynthesis/implant configuration

Two osteosynthetic systems for EADHFs with 3.5-mm mono-axial locking screws (DePuy Synthes, Oberdorf, Switzerland) were tested in this study as follows: six hole-ARPP and five hole-EADHP. Each plate was fixed with three bicortical locking screws in the proximal fragment and four bicortical locking screws in the distal fragment (Figures 1a,b). Care was taken to prevent the distal screws in the EADHP from penetrating the opposite cortex or joint surface by stopping the drill bit from pushing when the bony density (hard end) of the far cortex was encountered. All osteosythesess were performed by a single senior orthopaedic trauma surgeon (ApK) to minimize variability in plate positioning and screw insertion.

### 2.3 Potting

After osteosynthesis, all the composite humeri were cut at 190 mm from their proximal ends for fixation in the clamping device to avoid loosening. For the biomechanical test with torsional loading, the distal part of the composite bone was embedded in epoxy resin in a cuboidal steel pot to allow for unconstrained loading.

### 2.4 Mechanical testing

**FIGURE 2:**
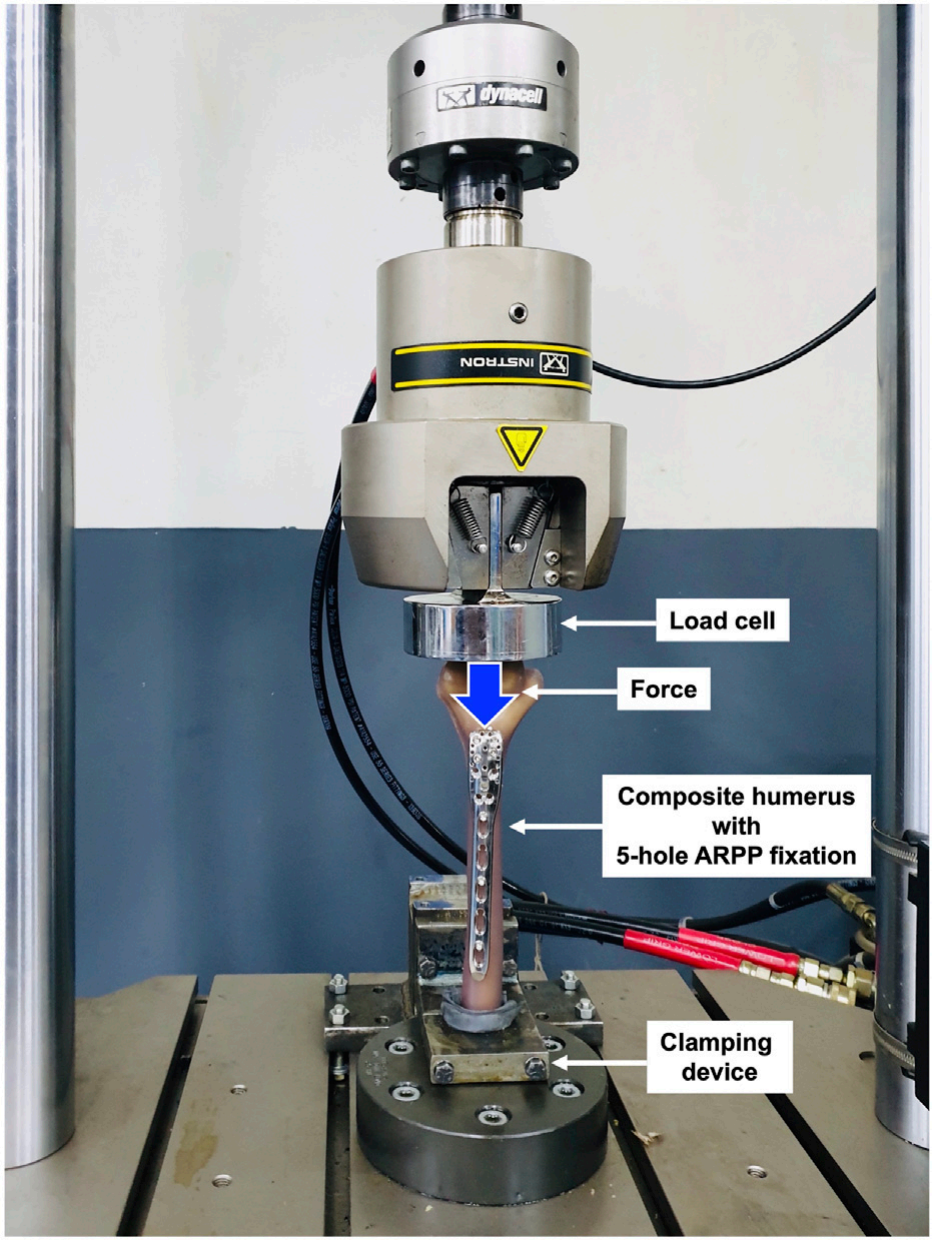
Setup for the biomechanical test. The composite humerus was cut 190 mm from the proximal end to allow fixation with a clamping device, enabling axial compression loading.

A mechanical testing machine (INSTRON 8872, Illinois Tool Works, Illinois, USA; Figure 2), calibrated according to the manufacturer’s guidelines before testing, was used to apply various loads to the specimens. Three specimens each for ARPP and EADHP underwent the stiffness test under anterior bending, axial compression, and torsion. The application of near-physiological loading conditions during the stiffness test was selected based on the protocols described by An et al. (1984); An et al. (1989) and Korner et al. (2004). In the axial compression and anterior bending tests, the corresponding destructive forces were applied to each construct. The measured forces were plotted against the deformation distances, and these plots were then used to determine the stiffness, which was the linear gradient of the graph in a range of forces from 0 to 120 N, and the failure load of the construct. Although the non-destructive torques at the maximum of 9 N m were applied on each construct initially, both internal and external torsional tests were used to determine the corresponding stiffness values (Figure 2). Both internal and external torsional forces were separately applied to quantify the stiffness on the same specimen. Therefore, only one load condition that was likely to be the common cause of this type of treatment failure was chosen for subsequent destructive tests. At the end of the torsional test, the internal torsional force was destructively applied to each specimen until failure of the construct was visually observed or the limit of the testing machine was reached to determine the failure load.

Failure was defined as the first abrupt change in the load–displacement curve associated with hardware pullout, fracture of the composite bone, or permanent plate deformation resulting in closure of the osteotomy gap. The failure load was defined as the magnitude of the load prior to an abrupt change in the plot between the deformation distances and measured loads. However, the curve may differ depending on the type of failure.

The load–deformation data were recorded using the INSTRON 8872 testing machine and exported as a comma-separated value file for subsequent analysis. Only three specimens per group were used for stiffness and load-to-failure testing, which limits statistical power and reduces the ability to generalize the findings. Therefore, the results should be interpreted with caution and regarded as preliminary.

### 2.5 Statistical analysis

Data and graphs were analysed using the R Software 3.5.1 (R Foundation for Statistical Computing). Statistical assumptions were verified using Shapiro-Wilk test for normality and Levene’s test for equal variances. If both assumptions were met, Student’s t-test was used to compare the average values of load-to-failure and stiffness in each group (EADHP and ARPP) under axial compression, anterior bending, and torsional loading. If assumptions were violated, Mann-Whitney U test was used as a non-parametric alternative. Effect sizes (Cohen’s d), 95% confidence intervals, and Bayes factors (BF10) were computed for all comparisons. Statistical significance was set at *p* < 0.05.

## 3 Results

The ARPP demonstrated significantly greater bending stiffness compared with the EADHP. In contrast, no significant differences were observed between these two groups in axial compression or torsional performance, with both constructs providing sufficient stability under these conditions. The detailed results were as follows.

### 3.1 Axial compression testing

**FIGURE 3:**
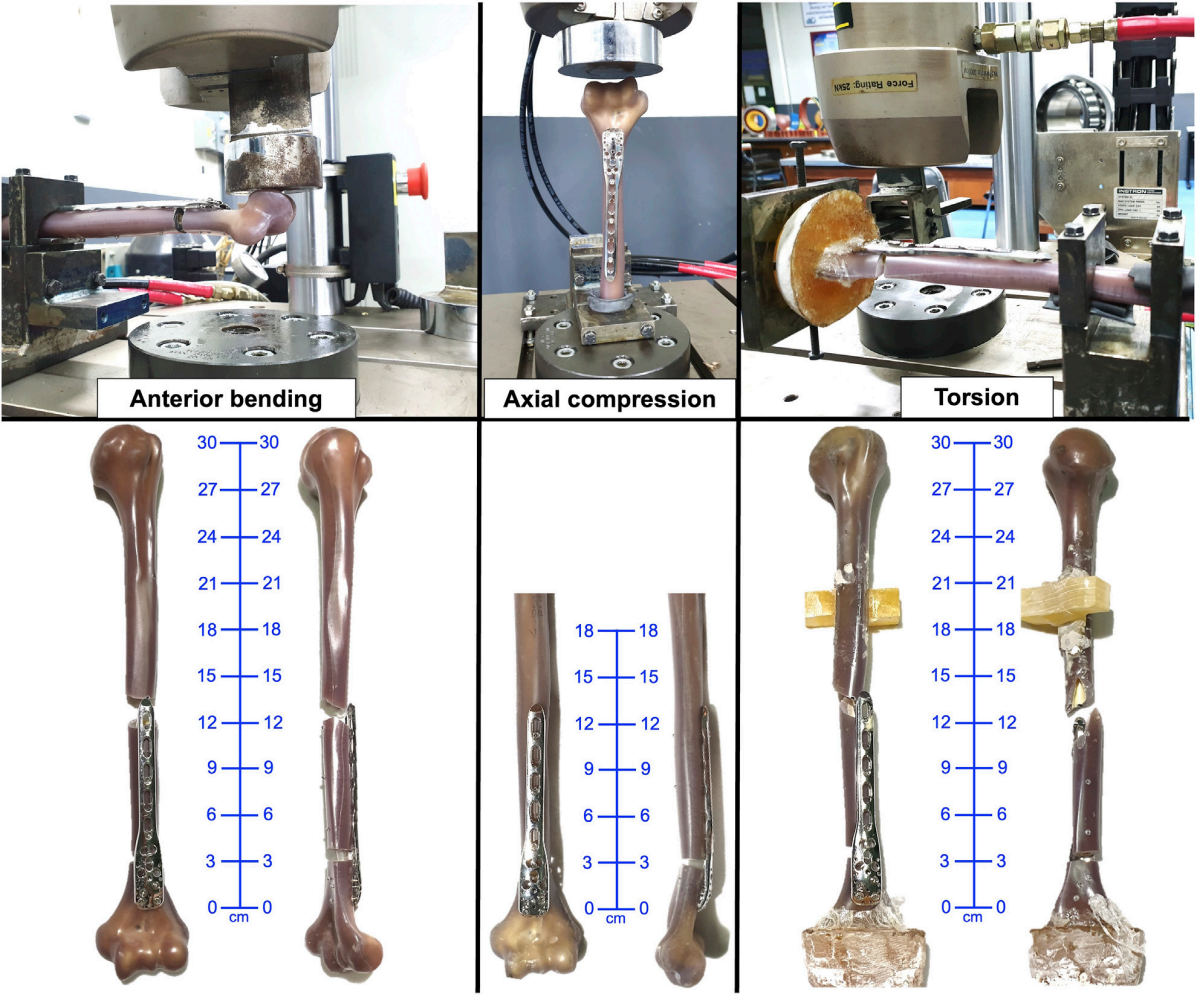
Failure of specimen in the ARPP group under anterior bending, axial, and torsional loading. ARPP, anterior reverse PHILOS plate.

**FIGURE 4:**
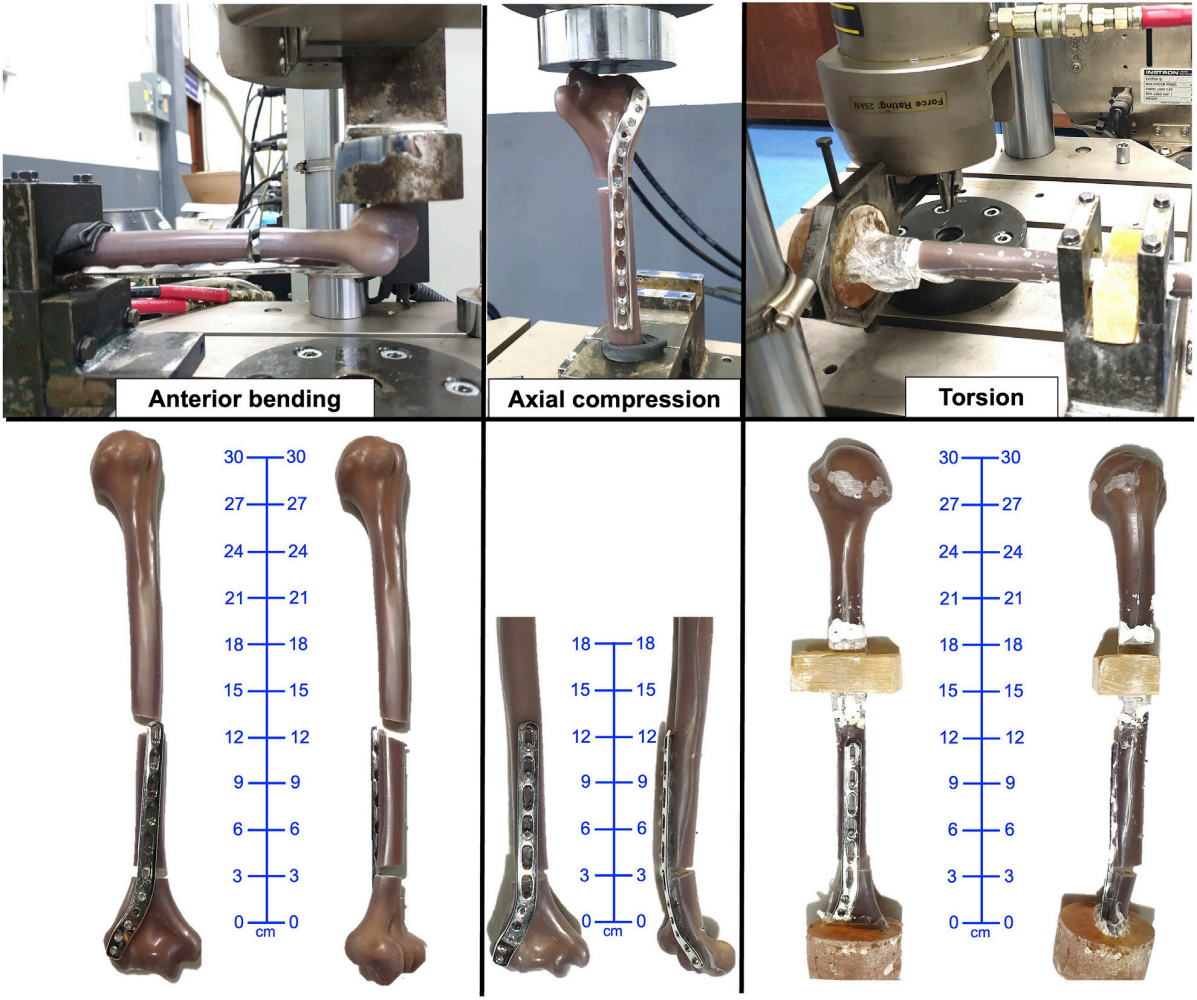
Failure of specimen in the EADHP group under anterior bending, axial, and torsional loading. EADHP, extra-articular distal humeral plate.

**FIGURE 5:**
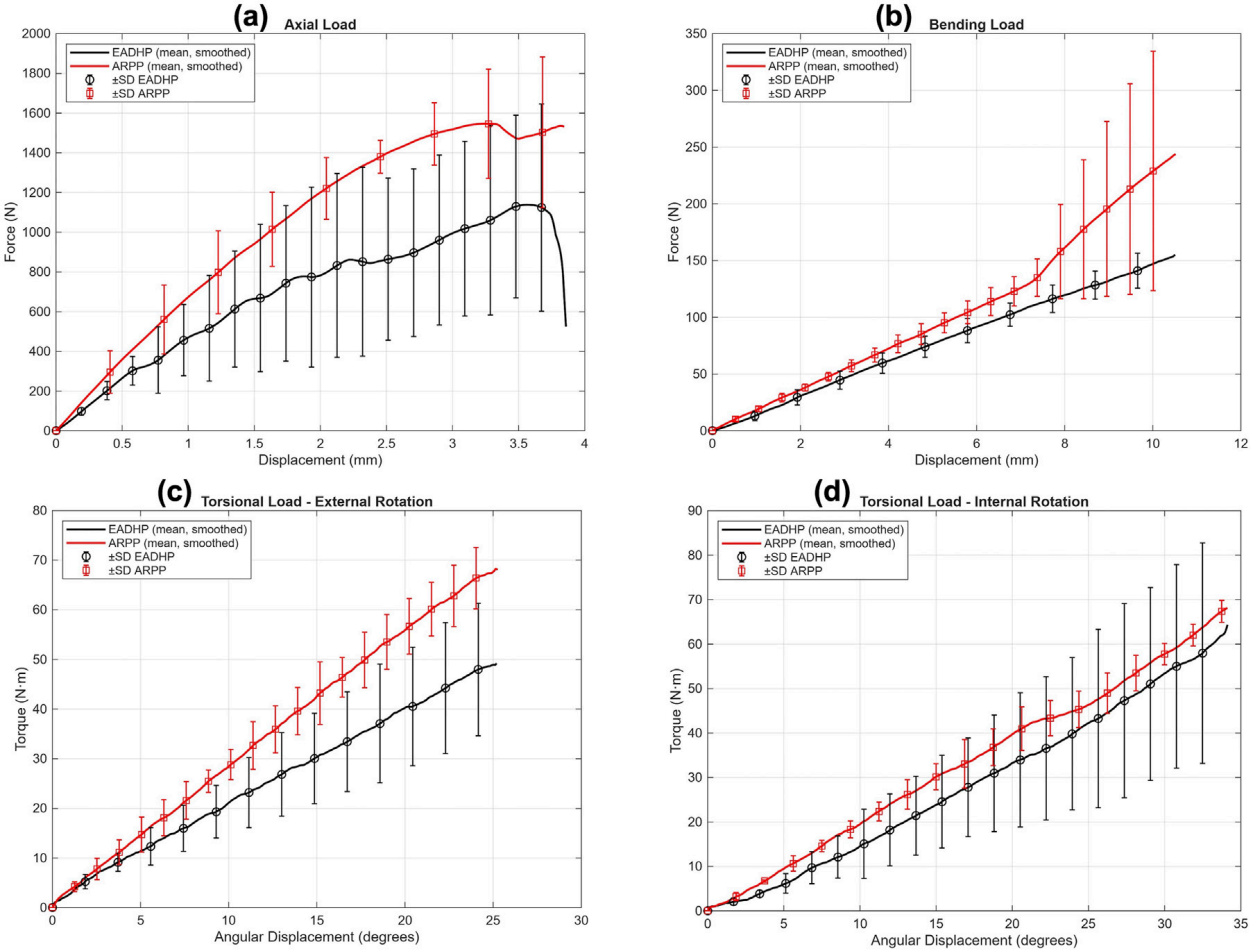
Average force-displacement relation of specimens under **(a)** axial compression, **(b)** anterior bending, and **(c,d)** torsional loading.

Failure in axial loading was indicated by closure of the osteotomy gap or plastic deformation of the EADHP or ARPP constructs. One specimen in the ARPP group sustained a fracture at the proximal plate–bone junction (Figures 3, 4). The ARPP group demonstrated higher average stiffness (668.91 ± 120.66 N/mm; 95% CI: 369.2–968.6) than the EADHP group (171.19 ± 45.44 N/mm; 95% CI: 58.3–284.1), although this difference was not statistically significant (*p* = 0.100, Mann–Whitney U, Cohen’s d = 5.46, BF10 = 737; Table 1). Similarly, the ARPP group exhibited greater maximal load-to-failure (2,092.56 ± 305.16 N; 95% CI: 1,334.5–2,850.6) compared with the EADHP group (907.03 ± 56.48 N; 95% CI: 766.7–1,047.3), but this difference also did not reach statistical significance (*p* = 0.100, Mann–Whitney U, Cohen’s d = 5.40, BF10 = 696; Table 1). The average force–displacement curves under axial compression are shown in Figure 5a.

**TABLE 1:** Stiffness and failure loads in the ARPP and EADHP groups during axial, bending, and torsional testing (*n* = 3 per group; values represent mean ± SD of three specimens per test condition).

Testing	EADHP Mean ± SD [95% CI]	ARPP Mean ± SD [95% CI]	*p*-value (test)	Cohen’s d	BF10	Conclusion
Axial load						
Stiffness (N/mm) Failure load (N)	171.19 ± 45.44 [58.3–284.1] 907.03 ± 56.48 [766.7–1,047.3]	668.91 ± 120.66 [369.2–968.6] 2,092.56 ± 305.16 [1,334.5–2,850.6]	0.100 (MW) 0.100 (MW)	5.46 5.40	737 696	Not Significant Not Significant
Bending load						
Stiffness (N/mm) Failure load (N)	13.93 ± 0.99 [11.5–16.4] 410.92 ± 75.81 [222.6–599.3]	17.78 ± 2.01 [12.8–22.8] 309.11 ± 67.66 [141.0–477.2]	0.041 (t) * 0.158 (t)	2.44 −1.42	14 2.20	Significant Not Significant
Torsional load						
External rotational stiffness (N·m/°) Internal rotational stiffness (N·m/°) Failure load (N·m)	0.46 ± 0.02 [0.4–0.5] 1.08 ± 0.42 [0.1–2.1] 26.03 ± 4.44 [15.0–37.1]	0.48 ± 0.02 [0.4–0.5] 1.15 ± 0.12 [0.8–1.5] 31.12 ± 0.84 [29.0–33.2]	0.444 (t) 0.797 (t) 0.100 (MW)	0.69 0.22 1.59	0.67 0.43 3.04	Not Significant Not Significant Not Significant

*Indicates significant difference.

ARPP, anterior reverse PHILOS, plate; EADHP, extra-articular distal humeral plate; SD, standard deviation; CI, confidence interval.

MW, Mann-Whitney U test; t, Student’s t-test; BF10, Bayes factor 10 (BIC).

### 3.2 Anterior bending testing

Failure during anterior bending occurred at the proximal plate–bone junction in either a transverse or short oblique configuration in both the ARPP and EADHP groups (Figures 3, 4). The ARPP group demonstrated significantly higher average stiffness (17.78 ± 2.01 N/mm; 95% CI: 12.8–22.8) compared with the EADHP group (13.93 ± 0.99 N/mm; 95% CI: 11.5–16.4) (*p* = 0.041, t-test, Cohen’s d = 2.44, BF10 = 14; Table 1). In contrast, the EADHP group showed a higher average maximal load-to-failure (410.92 ± 75.81 N; 95% CI: 222.6–599.3) compared with the ARPP group (309.11 ± 67.66 N; 95% CI: 141.0–477.2), although the difference was not statistically significant (*p* = 0.158, t-test, Cohen’s d = −1.42, BF10 = 2.2; Table 1). The average force–displacement curves under anterior bending are shown in Figure 5b.

### 3.3 Torsional testing

Failure of the construct occurred as a spiral fracture at the proximal plate–bone junction in the ARPP group, whereas the EADHP group exhibited plastic deformation of the plate (Figures 3, 4). The average torque–displacement curves under external and internal rotation are shown in Figures 5c,d.

The ARPP group demonstrated slightly higher average stiffness in external rotation (0.48 ± 0.02 N m/degree; 95% CI: 0.4–0.5) compared with the EADHP group (0.46 ± 0.02 N m/degree; 95% CI: 0.4–0.5), but the difference was not statistically significant (*p* = 0.444, t-test, Cohen’s d = 0.69, BF10 = 0.67; Table 1). Similarly, the ARPP group showed higher average stiffness in internal rotation (1.15 ± 0.12 N m/degree; 95% CI: 0.8–1.5) than the EADHP group (1.08 ± 0.42 N m/degree; 95% CI: 0.1–2.1), although the difference was not significant (*p* = 0.797, t-test, Cohen’s d = 0.22, BF10 = 0.43; Table 1). The ARPP group also demonstrated a higher average torsional load-to-failure (31.12 ± 0.84 N m; 95% CI: 29.0–33.2) compared with the EADHP group (26.03 ± 4.44 N m; 95% CI: 15.0–37.1), but again, the difference was not statistically significant (*p* = 0.100, Mann–Whitney U, Cohen’s d = 1.59, BF10 = 3.04; Table 1).

## 4 Discussion

EADHF is a technically challenging injury to manage because of the difficulty in establishing adequate fixation in a short distal fragment and the potential risk of iatrogenic radial nerve injury. Clinically, ARPP via the anterior approach is considered an alternative for treating such fractures (Park et al., 2015; Sohn and Shin, 2019; Jitprapaikulsarn et al., 2020). In our study, the biomechanical performance of ARPP was largely equivalent to that of the commonly used implant EADHP, with superiority only in bending stiffness (mean 17.78 ± 2.01 N/mm vs 13.93 ± 0.99 N/mm; *p* = 0.041, Cohen’s d = 2.44, BF10 = 14), suggesting enhanced resistance to anterior-posterior loads that mimic early mobilization stresses. This aligns with prior studies showing ARPP’s multidirectional locking screws improve rotational stability in metaphyseal regions (Jitprapaikulsarn et al., 2020).

Notably, large non-significant differences in axial failure load (ARPP: 2,092.56 ± 305.16 N [95% CI: 1,334.5–2,850.6] vs EADHP: 907.03 ± 56.48 N [95% CI: 766.7–1,047.3]; Cohen’s d = 5.40, BF10 = 696) provide strong Bayesian evidence for a difference, though frequentist *p* = 0.100 and wide 95% CIs reflect uncertainty from small sample size (*n* = 3). Such effects may hold clinical relevance in active patients, where higher axial capacity could reduce failure risk under compressive loads (Lee et al., 2023). Similarly, the 19.55% higher torsional failure load for ARPP (Cohen’s d = 1.59, BF10 = 3.04) might mitigate rotational failures during daily activities (Lee et al., 2023), though inconclusive due to non-normality (Shapiro-Wilk *p* = 0.038 for EADHP).

To date, the optimal treatment for EADHFs remains debatable. Several studies have demonstrated that open reduction and internal fixation with posterior plating are reliable options (Capo et al., 2014; Illical et al., 2014; Gallucci et al., 2015; Kharbanda et al., 2017; Ali et al., 2018). Double- and single-column plate osteosynthesis have been applied. Although dual plating provides biomechanically stronger fixation (Tejwani et al., 2009), the higher risk of infection and delayed/nonunion from more extensive soft tissue stripping and longer operative times are the major drawbacks of this type of fixation (El Mahboub and Arafat, 2012; Kharbanda et al., 2017). Single-column plating has gained popularity for reducing the adverse consequences of dual plating. A combination of one or two 3.5-mm lag screws and a single 4.5-mm locking compression plate is an acceptable option for treating distal diaphyseal fractures of the humerus. To achieve sufficient stability, at least two locking screws should be used in the distal fragment. For implant positioning, the distal end of the plate must be placed proximal to the roof of the olecranon fossa. Therefore, this fixation technique should be avoided in cases of lower distal diaphyseal fractures, particularly in osteoporotic bones with severe comminution (Kumar et al., 2015).

Anatomically pre-contoured locking compression plate or EADHP has been used for stabilising a particular fracture location in the distal humeral shaft. This specifically designed device can be placed distally to the most distal part of the lateral column, facilitating 5-locking screw fixation in the distal fragment (Ali et al., 2018). Nevertheless, the higher risk of radial nerve injury and a decrease in muscular strength due to the use of the triceps-splitting approach are major complications of the posterior plating technique (Capo et al., 2014; Illical et al., 2014; Gallucci et al., 2015; Kharbanda et al., 2017; Ali et al., 2018; Jiamton et al., 2019). In addition, Zhou et al. (2014) demonstrated a mismatch of the EADHP in the Chinese population, especially when a three-to six-hole plate was used.

Recent studies delineated the utility of ARPP via the anterior approach for the treatment of EADHFs (Park et al., 2015; Lim et al., 2020; Lee et al., 2023; Fu et al., 2025). The remarkable advantages of this method include a lower risk of radial nerve injury and the ability to apply a cluster of multidirectional locking screws to the short distal fragments of the humerus. However, it is important to note that our biomechanical study did not directly evaluate radial nerve safety. Therefore, any clinical consideration regarding reduced nerve injury risk should be regarded as speculation derived from previous reports, rather than as a finding of the present study.

The present biomechanical analysis is a preliminary study comparing ARPP and EADHP as osteosynthetic techniques for the treatment of EADHFs. The biomechanical setup used in this study employed extended bending, axial compression, and torsional forces to represent the physiological loading conditions of the elbow joint. An et al. (1984) demonstrated that the resultant joint force occurring at various positions of the elbow joint under normal daily activities, including weight lifting, was approximately 0.3–0.5 times the body weight, which was equivalent to 210–350 N for a 70-kg person. In the present study, both the EADHP and PHILOS provided adequate joint stability for the forces in this range.

The positioning of distal cortical screws differs between ARPP and EADHP, influencing construct stability and likely contributing to the observed differences in stiffness. Because ARPP is centered in the distal humeral metaphysis and allows multidirectional screw fixation into both the medial and lateral columns, it provides more balanced force distribution than EADHP. In this study, ARPP demonstrated significantly greater stiffness during anterior bending tests and higher average load-to-failure values under axial and torsional loading. By contrast, the distal end of EADHP, positioned more distally but only laterally on the distal condyle, may explain its relatively lower stiffness across all testing modes. These findings align with the computational work of Jitprapaikulsarn et al. (2024), who demonstrated the biomechanical advantages of ARPP over EADHP in terms of lower Equivalent von Mises stress.

This study has several limitations. The small sample size reduced the statistical power to detect significant differences. Although synthetic composite bones ensured standardization and reproducibility, they do not reflect the variability in bone density, geometry, fracture morphology, or the influence of surrounding soft tissues found in real patients. This is considered the strongest limitation and substantially reduces the direct clinical applicability of the findings. The use of only one fracture type (AO/OTA 12-C3) further restricts generalizability to other patterns, such as osteoporotic or less comminuted fractures. Testing was limited to monotonic load-to-failure, without cyclic or fatigue loading to simulate repetitive physiological stresses. Finally, all fixations were performed by a single surgeon to minimize performance bias, but this does not account for inter-surgeon variability in clinical practice.

## 5 Conclusion

The ARPP demonstrated superior bending stiffness compared with the EADHP, while showing equivalent performance in axial compression and torsional parameters. These findings suggest that ARPP may represent a promising alternative construct for the fixation of extra-articular distal humeral fractures. However, it should be emphasized that our results reflect mechanical performance in a controlled experimental setting rather than broad clinical outcomes. Therefore, further investigation to clinical practice should be made with caution, and further validation using cadaveric models and clinical studies is necessary.

## Data Availability

The original contributions presented in the study are included in the article/supplementary material, further inquiries can be directed to the corresponding author.

## Abbreviations

ARPP, anterior reverse PHILOS plate; EADHF, extra-articular distal humeral fracture; EADHP, extra-articular distal humeral plate; PHILOS, proximal humeral internal locking system.
